# Family Resilience Scale Short Form (FRS16): Validation in the US and Chinese Samples

**DOI:** 10.3389/fpsyt.2022.845803

**Published:** 2022-05-13

**Authors:** Tak Sang Chow, Catherine So Kum Tang, Tiffany Sok U. Siu, Helen Sin Hang Kwok

**Affiliations:** ^1^Department of Counselling and Psychology, Hong Kong Shue Yan University, North Point, Hong Kong SAR, China; ^2^Wan Chow Yuk Fan Centre for Interdisciplinary Evidence-based Practice and Research, Hong Kong Shue Yan University, North Point, Hong Kong SAR, China

**Keywords:** family resilience, short scale, Chinese, US, measurement validation

## Abstract

Family resilience, which refers to the processes through which a family adapts to and thrives from adversities, has growing importance in recent years. In response to the need for further research on family resilience, the present research aims to abbreviate and validate Sixbey's Family Resilience Assessment Scale (FRAS) into a 16-item version Family Resilience Scale Short Form in the US (FRS16) and Chinese (FRS16_C) samples. The samples included 1,236 (Study 1) and 1,135 (Study 2) participants from the US and China, respectively. Results of confirmatory factor analysis (CFA) supported the proposed three-factor structure of FRS16: Family Communication and Connectedness, Positive Framing, and External Support across two samples. Overall, the reliability and validity of full and subscales of FRS16 and FRS16_C were satisfactory. Multi-group CFA revealed that both configural and metric invariance are supported, suggesting that participants in the US and Chinese samples assign comparable meaning to the latent factors of FRS16. Results suggested that FRS16 and FRS16_C are valid instruments for family resilience in the US and Chinese samples.

## Introduction

Since 1990s, the term “family resilience” has emerged in family sciences to signify family processes that create perseverance, accommodation, and growth in responses to crises and challenges (1–3). The conceptualization of family resilience entails two important features. First, family resilience is more than the average of individual members' resilience, but rather encompasses dynamic, structural features of the family system which could only be understood through a broader, relational framework (4). Second, it involves more than the capacity to survive a crisis, but also to realize the potential for growth out of difficulties (3). Overcoming a challenge together, a family can emerge as more resourceful, connected, and loving in facing future crises.

### Walsh's Family Resilience Model

Building upon earlier strength-based family paradigms which emphasize the interaction between stressors, family perceptions and protective factors in developing resilience (e.g., The Double ABCX model; (5), Walsh (3) proposed a family resilience model that specifies three overarching processes that promote family resilience, each of which consists of several subprocesses. The three overarching processes are ***Family Belief Systems***, ***Organizational*** ***Patterns***, and ***Communication Processes***. Family Belief Systems refer to the shared reality that might facilitate normalizing and overcoming a crisis via meaning-making, maintaining positivity, and a transcendent, spiritual grounding. Organizational Patterns refer to the ways families operate during a crisis. Lastly, Communication Processes involve clear communication, open emotional expression, and collaborative problem-solving. In this framework, family resilience is a dynamic process that varies across families, contexts, and time.

This family resilience framework has tremendous implications on how to successfully cope with abrupt and persistent crises including caregiving for a member with chronic illnesses (6), traumatic societal events such as natural disaster (7) and pandemic (8), divorce (9), and the death of a family member (10). Importantly, family resilience influences children's positive development (11, 12). Thus, prevention and intervention programs have been developed to identify and foster family resilience, in the hope of preserving family functioning and well-being during difficult situations [e.g., (13–15)].

### The Family Resilience Assessment Scale (FRAS)

In view of the theoretical importance of the family resilience construct and the lack of quantitative measurement of it, Sixbey (16) developed the Family Resilience Assessment Scale (FRAS). The 54-item FRAS was developed upon Walsh (3)'s family resilience model. It includes six subscales, which reflect Walsh's three overarching family resilience processes (Family Belief Systems, Organization Patterns, and Communication Processes). Family Belief Systems are associated with the subscales *Maintaining a Positive Outlook, Ability to Make Meaning of Adversity*, and *Family Spirituality*. Organization Patterns are associated with two subscales, *Utilizing Social and Economic Resources* and *Family Connectedness*. Finally, Communication Processes are associated with the subscale *Family Communication and Problem Solving*.

Ever since its birth, researchers have observed the predictive value of the FRAS on mental health measures. For instance, higher score on the FRAS was associated with lower depression, anxiety, stress (17), and higher individual resilience (18, 19). It also served as a protective factor for cancer patients and their spouses (20) as well as young people with severe epilepsy (21). Relevant to the current context, it also mitigated the negative effect of pandemic-related stressors on stress severity (17). Thus, far, the FRAS has been translated to different languages such as Chinese (22–24), Turkish (25), and Polish (26).

### The Need of a Brief Measure of Family Resilience

The original FRAS consists of 54 items. Its length makes it cumbersome as a process or an outcome measure especially in studies that involve clinical trials, or longitudinal mutli-wave investigation, as researchers are faced with limited assessment time. A brief measure of family resilience is needed to advance the research of family resilience. To our best understanding, the shortest form of FRAS was the Chinese version which still contains 32 items (24), while most other versions have more than 40 items (e.g., (22)). Furthermore, recent studies reveal the possibility of a more parsimonious factor structure of the FRAS. While Kaya and Arici (25) found a 4-factor solution, a 3-factor model showed adequate fit in Li et al. (24)'s study.

As suggested by Burisch (27), acceptable validities and reliabilities can be achieved by a fairly small number of items. The present study proposed a 3-factor model which is built upon Li et al. (24)'s findings. The three proposed factors are (1) ***Communication and*** ***Connectedness***; (2) ***Positive Framing***; and (3) ***External Resources***. The first factor combines the Family Communication and Family Connectedness factors in the original scale as effective family communication can only be achieved with an optimal balance of mutual support and respect of individuals' autonomy (28). These two elements are theoretically intertwined. The second factor, ***Positive framing***, refers to the attitudes or beliefs that help a family to maintain positivity during adversities. It combines the subcomponents Maintaining a Positive Outlook and Making Meaning of Adversities in the original FRAS which are two crucial components across different family resilience conceptualizations (29, 30). ***External Resources*** are important for families to cope with internal and external crises. Across the literature, social [e.g., neighbor; (31)], economic [e.g., welfare system; (32)], and spiritual [e.g., church or other religious institutions; (33)] resources are important external support for family to function well during crises. Thus, this factor captures the subcomponents Utilizing Social and Economic Resources and Family Spirituality in the original scale. Therefore, the present studies aim to derive a short scale of family resilience with a more parsimonious 3-factor structure to make family resilience research more feasible and less time-consuming. We started with selecting essential items which have the highest factor loadings from the original FRAS and then examined its psychometric properties in a US and then in a Chinese sample. Since past studies found that family resilience was associated with relationship functioning and well-being, the FRAS is hypothesized to be associated with family cohesion (34), relationship satisfaction (35), general health (36), quality of life (37), and perceived community support (38). Overall, the current research aims to abbreviate and validate Sixbey's Family Resilience Assessment Scale (FRAS) into a shorter form and cross-validated it in both the US (study 1) and Chinese (study 2) samples.

## Study 1

Study 1 aims to derive a reliable and valid short scale to measure family resilience based on FRAS in the US sample. We will first identify the most essential items of FRAS according to factor loadings and item-to-subscale correlation. Then, a factorial structure will be identified with CFA. Furthermore, its associations with related variables and the reliability of the composite and subscales will also be examined.

## Method

### Procedure

We recruited participants from the US via Amazon's Mechanical Turk (MTurk), a commonly used online crowdsourcing platform that has a diverse and stable subject pool (39). Past studies suggested that participants recruited from MTurk is more representative of the US sample in comparison to face-to-face convenience samples (40). Consent form was presented on the first page of the online survey, participants indicated understanding of their rights and granted consent by pressing “continue to next page.” Participants' confidentiality was guaranteed that personal identities are not traceable, and the data will only be used for research purposes. After data exportation from the platform, data will be stored in a local drive and is solely accessible to members of the research team. Participants must be US citizens aged above 18. They were given 1 week to complete the survey and were compensated with 0.5 USD through the built-in payment system in MTurk upon completion. This study was approved by the Human Research Ethics Committee of the affiliated university of the research team.

### Characteristics of Participants

After excluding respondents who provided statistical improbable answers (e.g., worked for 500 h on average per week in the past 12 months) and did not provide answers to the key variables (i.e., family resilience and validation instruments), 1,236 participants were available for the CFA analysis. The average age of participants was 36.17 (*SD* = 10.83, range = 18 to 75), and 63.7% were male. The majority of them were highly educated (92.1 % obtained a bachelor's degree or above), married (79.4%), and with an annual household income of $40,000 or above (71.1%) (see Table 1).

**Table 1:** Descriptive statistics of demographics of US sample.

	***n***	**%**	***M* (*SD*)**	**Range**
Demographic variable				
Gender				
Male	787	63.7		
Female	449	36.3		
Age (in years)			36.17 (10.83)	18–75
Annual household income (USD)				
< $10,000	44	3.6		
$10,000 to $39,999	313	25.3		
$40,000 to $79,999	627	50.7		
$80,000 to $119,999	217	17.6		
≥$120,000	35	2.8		
Past year employment status				
Full-time/part-time	1,209	97.8		
Unemployed/retired	27	2.2		
Average weekly working hours			36.52 (12.33)	0–100
Relationship status				
Single	208	16.8		
Married	982	79.4		
Separated/divorced/widowed	11	0.9		
In a relationship	35	2.8		
Education level				
Secondary school or below	32	2.6		
Associate/Diploma	66	5.3		
Bachelor's degree	921	74.5		
Postgraduate degree	217	17.6		
Religious belief				
Christian	665	53.8		
Catholic	426	34.5		
Islamic	15	1.2		
Jewish	24	1.9		
Buddhist	5	0.4		
Hindu	26	2.1		
No religious belief	68	5.5		
Others	7	0.6		

### Measures

#### Family Resilience Scale Short Form (FRS16)

First, we selected essential items for the current proposed **Family Resilience Scale Short Form** from Family Resilience Assessment Scale (FRAS) (16). Considering the original scale has disproportionately large numbers of items in the Family Communication and Problem Solving (FCPS) subscale, the items of our proposed scale were selected with two criteria based on Sixbey's findings in 2005: (1) Two items with the highest factor loadings for each subscale (except for FCPS) (2) For FCPS, 6 items with both factor loadings and item-to-subscale correlation higher than 0.7. As a result, 16 items were selected for the short form (see Appendix A). Thus, the abbreviation for Family Resilience Scale Short Form will be FRS16. Participants indicated their agreement with 16 statements on a 4-point scale (1 = *strongly disagree* to 4 = *strongly agree*).

#### Validation Instruments

**Quality of life** was accessed with the 8-item EUROHIS-QOL scale which has been validated across different cultures (41). Participants indicated their satisfaction with different aspects of their lives on a 5-point scale (1 = *not at all*, 5 = *completely*).

**General health** was accessed with the General Health Questionnaire-12 [GHQ-12; (42)] which is intended to tap on respondents' recent functioning such as mood, sleeping quality and concentration compared to their normal state. The items are rated on a 4-point scale (1 = *less than usual*, 4 = *much more than usual*), and scores were summed across items with a higher score reflecting worse health.

**Relationship satisfaction** was accessed with the 7-item Relationship Assessment Scale [RAS; (43)]. Participants indicated how satisfied they were with their partner and relationship based on a 5-point scale (1 = *low satisfaction*, 5 = *high satisfaction*).

**Family cohesion** was accessed with the 9-item family cohesion subscale of the Family Environment Scale (44). Participants indicated their agreement with the statements that described their family (0 = *disagree*, 1 = *agree*), with a higher summation score reflecting higher family cohesion.

**Perceived community support** was assessed with the 2-item integration and need fulfillment subscale of the Brief Sense of Community Scale (45) with a 5-point scale (1 = *strongly disagree*, 5 = *strongly agree*) to reflect the degree to which participants felt supported by their community.

## Results

The descriptive statistics for demographic and main variables are summarized in Tables 1, 2, respectively.

**Table 2:** Descriptive statistics of main variables of US sample.

		**α**	***M* (*SD*)**	**Range**	**Bivariate correlations**			
	**FRS16**				**1**	**2**	**3**	
1	Full scale	0.87	49.41(7.24)	16–64				
2	Communication and Connectedness	0.78	24.81(3.83)	8–32	0.828^**^			
3	Positive Framing	0.64	12.43(2.13)	4–16	0.939^**^	0.697^**^		
4	External Resources	0.66	12.16(2.32)	4–16	0.809^**^	0.514^**^	0.637^**^	
	**Validation instruments**				**1**	**2**	**3**	**4**
5	Quality of life	0.84	3.86(0.64)	1–5	0.664^**^	0.534^**^	0.627^**^	0.544^**^
6	General health	0.53	16.89(4.27)	0–35	−0.163^**^	−0.125^**^	−0.152^**^	−0.142^**^
7	Family cohesion	0.42	5.63(1.76)	0–9	0.294^**^	0.254^**^	0.309^**^	0.173^**^
8	Relationship satisfaction	0.57	3.52(0.52)	1–5	0.291^**^	0.253^**^	0.303^**^	0.165^**^
9	Perceived community support	0.53	3.83(0.77)	1–5	0.588^**^	0.438^**^	0.570^**^	0.487^**^

** *p <0.01*.

### Confirmatory Factor Analysis (CFA) of FRS16

**Figure 1:**
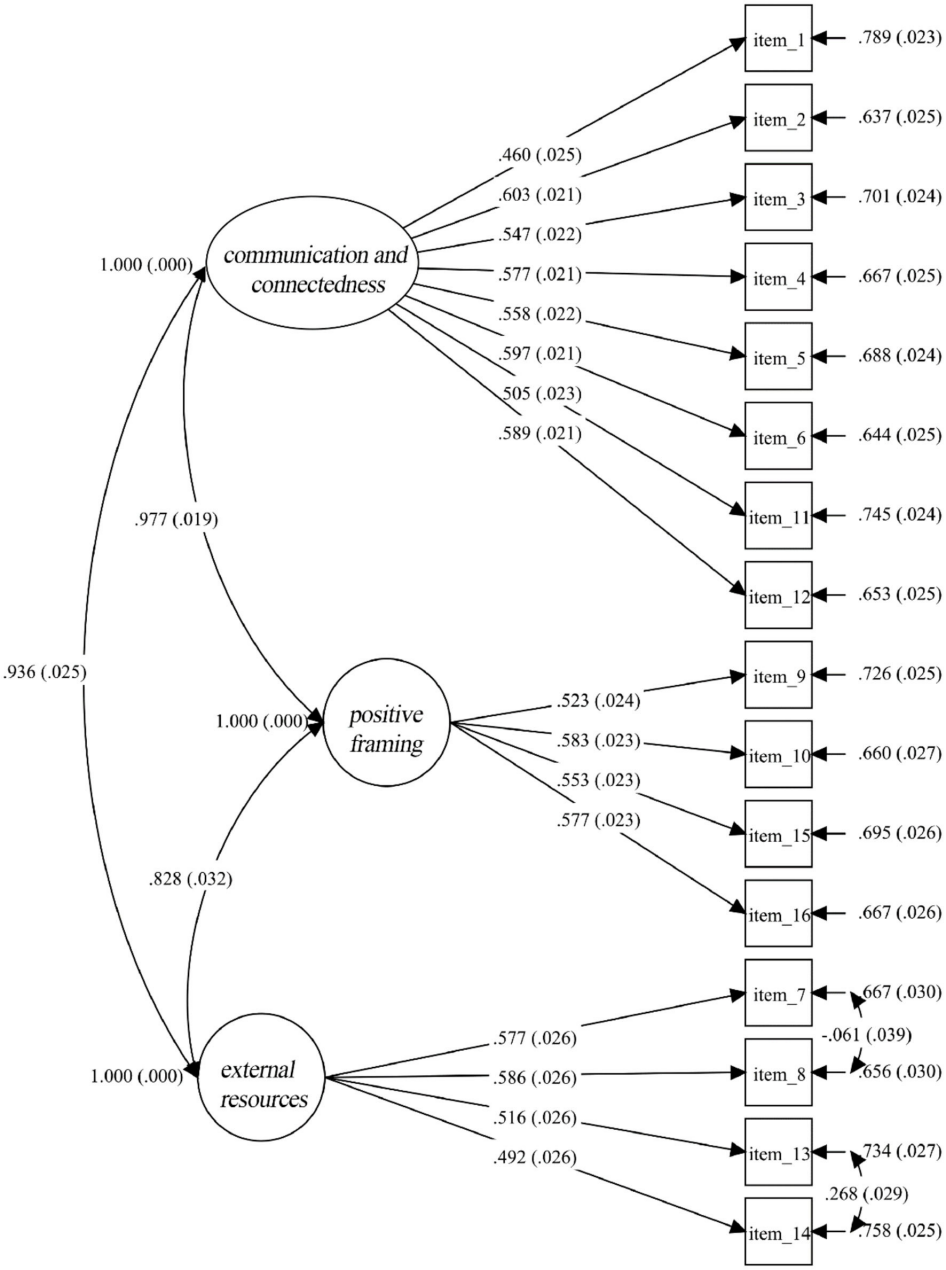
Factor structure and standardized factor loadings of FRS16.

The factor structure of the proposed three-factor model was assessed with CFA. CFA was performed with the maximum likelihood estimation in Mplus 8.3 (46). As suggested by the CFA results, the proposed three-factor model has reasonable model fit: χ^2^ (99) = 699.15, *p* <0.001; SRMR = 0.046; RMSEA = 0.070, CI = [0.065, 0.075]; TLI = 0.86; CFI = 0.88. The factor solution and standardized factor-loadings of all items are shown in Figure 1. Additionally, four theoretically meaningful alternative models were also examined. In particular, the single-factor model assumes only one underlying latent factor of family resilience. The two-factor model distinguishes items into either external or internal resilience factors. The five-factor model involves problem solving, social and economic resources, positivity, family spiritually, and communication and connectedness. While the six-factor model is the one proposed in the original FRAS. As shown in Table 3, the fit indices improved as the number of factors increased in the model, but this trend slowed down when the number of factors reached to three. Since there were no significant differences in fit indices, the three-factor model seems to have the best balance of parsimony and interpretability of item clustering.

**Table 3:** Model fit indices of models with different number of factors.

**Model**	***df***	**χ^2^**	**CFI**	**TLI**	**RMSEA**	**SRMR**	**AIC**	**BIC**
One-factor	104	837.43	0.857	0.835	0.076	0.050	41845.14	42090.88
Two-factor	103	790.63	0.866	0.843	0.073	0.049	41800.34	42051.21
Three-factor	99	699.15	0.883	0.858	0.070	0.046	41716.87	41988.21
Fix-factor	94	659.73	0.889	0.859	0.068	0.045	41687.45	41984.38
Six-factor	89	560.76	0.908	0.876	0.065	0.042	41598.48	41921.01

### Validity and Reliability

Content validity was examined with the item-to-scale and item-to-subscale correlations. As shown in Table 4, all items were significantly and moderately correlated to both its subscale and full scale (*r*s ≥ 0.36).

**Table 4:** Item-to-scale and item-to-subscale correlations.

**Item**	**CC**	**PF**	**ER**	**FRS16**
1	0.357^**^			0.503^**^
2	0.452^**^			0.634^**^
3	0.468^**^			0.582^**^
4	0.457^**^			0.607^**^
5	0.450^**^			0.602^**^
6	0.461^**^			0.625^**^
11	0.405^**^			0.550^**^
12	0.449^**^			0.609^**^
9		0.470^**^		0.562^**^
10		0.487^**^		0.587^**^
15		0.485^**^		0.564^**^
16		0.494^**^		0.588^**^
7			0.649^**^	0.581^**^
8			0.665^**^	0.580^**^
13			0.753^**^	0.563^**^
14			0.731^**^	0.550^**^

** *p <0.01, CC, Communication and Connectedness; PF, Positive Framing; ER, External Resources; FRS16, Family Resilience Short Scale*.

We also examined whether FRS16 is associated with quality of life, general life and family cohesion, relationship satisfaction and perceived community support. As shown in Table 2, all of the subscales and the full scale of FRS16 were correlated with the proposed variables significantly the expected directions. Whereas, reliabilities of each subscale were above 0.6 and the reliability for the overall FRS16 scale was satisfactory.

## Study 2

Next, we aimed to cross-validate FRS16 with a Chinese sample which was abbreviated as FRS16_C. We first examined the construct validity of the three-factor structure in a Chinese sample with CFA, and conducted content validity tests and examination of the associations between FRS16_C and important outcomes. Additionally, the correlation patterns of the original and two other Chinese versions of FRAS and FRS16_C were examined to evaluate whether they are comparable instruments. Furthermore, test-retest reliability was also examined in a 2-week follow-up. Lastly, measurement invariance analysis was followed to obtain a stronger support that FRS16 operates similarly for both the US and Chinese samples, and participants from the two regions share parallel understandings of the construct.

## Method

### Procedure and Participants

Procedures were similar to that of Study 1, we recruited participants from mainland China via an online survey distribution platform Credamo (www.credamo.com) which is a professional research data platform with survey distribution and data modeling services. Datasets collected from this platform has been published in well-respected international journals such as Psychological Science and Journal of Consumer Research^1^. Credamo has over 2 million registered users cover all provincial regions in China which provides researchers a wider reach to participants in different regions within China. Upon completion, participants received 16 CNY in their Credamo account which can be transferred to his or her online payment platform WeChat Pay.

After excluding those who were not residing in mainland China, the final sample consisted of 1,135 participants for the CFA test. On average, participants aged 30.01 (*SD* = 5.99, range = 18–59) with slightly higher proportion of female (57.4%). The majority of them were married (68.8%), highly educated (85.6% with a bachelor's degree or above), and with a high annual household income within mainland China^2^ (61%) (see Table 5).

**Table 5:** Descriptive statistics of demographics (Chinese sample).

	**%**	***M* (*SD*)**	**Range**
Demographic variable			
Gender			
Male	42.6		
Female	57.4		
Age (in years)		30.01 (5.99)	18–59
Annual household income (CYN)			
≤ 7,380	1.8		
7,381–15,777	7.6		
15,778–25,035	10.9		
25,036–39,231	7.7		
25,036–76,401	11.1		
≥76,402	61.0		
Employment status in past year			
Full-time/part-time	99.2		
Unemployed	0.7		
Retired	0.1		
Average weekly working hours in the past year		41.64 (10.69)	0–85
Relationship status			
Single	20.4		
Married	68.8		
In a relationship	10.7		
Separated/divorced	0.1		
Year in marriage		6.65 (4.71)	1–36
No. of children		0.78 (0.65)	0–5
Education level			
Secondary school or below	4.4		
Associate/Diploma	10.0		
Bachelor's degree	73.4		
Postgraduate degree	12.2		
Religious belief			
Christian	2.2		
Catholic	0.1		
Buddhist	9.6		
No religious belief	87.5		
Others	0.6		

### Measures

This study included available Chinese translations of measures in Study 1. A list of validation studies on these Chinese versions of the measurement scales is available from the corresponding author upon request. In particular, the FRS16_C used the Chinese translation of FRAS by Chiu et al. (22) and refined some wordings to fit the context of mainland China.

All participants completed the full set of questionnaires, including the FRS16_C. Among them, 213 participants filled in the full version of FRAS to test the associations between FRS16_C with the original and two Chinese versions of FRAS and compare their correlations with different demographic and convergent variables as composite scores. Another subsample of 63 participants filled in the same set of measurements 2 weeks later for the temporal consistency test of FRS16_C.

## Results

All descriptive statistics for demographic and main variables are summarized in Tables 5, 6, respectively.

**Table 6:** Descriptive statistics of main variables (Chinese sample).

		***a***	***M* (*SD*)**	**Range**	**Bivariate correlations**			
	**FRS16_C**				**1**	**2**	**3**	
1	Full scale	0.78	49.38 (4.88)	24–62				
2	Communication and Connectedness	0.67	26.34 (2.67)	11–32	0.857^**^			
3	Positive Framing	0.51	13.17 (1.36)	5–16	0.768^**^	0.600^**^		
4	External Resources	0.68	9.87 (2.15)	4–16	0.719^**^	0.324^**^	0.367^**^	
	**Validation instruments**				**1**	**2**	**3**	**4**
5	Quality of life	0.86	3.98 (0.57)	1.63–5	0.589^**^	0.512^**^	0.513^**^	0.375^**^
6	General health	0.84	10.84 (5.45)	0–33	−0.549^**^	−0.449^**^	−0.460^**^	−0.397^**^
7	Family cohesion	0.70	8.07 (1.26)	0–9	0.410^**^	0.421^**^	0.322^**^	0.204^**^
8	Relationship satisfaction	0.78	4.40 (0.48)	1.71–5	0.437^**^	0.374^**^	0.389^**^	0.252^**^
9	Perceived community support	0.74	3.97 (0.65)	1–5	0.566^**^	0.430^**^	0.492^**^	0.440^**^

** *p <0.01*.

### Confirmatory Factor Analysis (CFA) for FRS16_C

**Figure 2:**
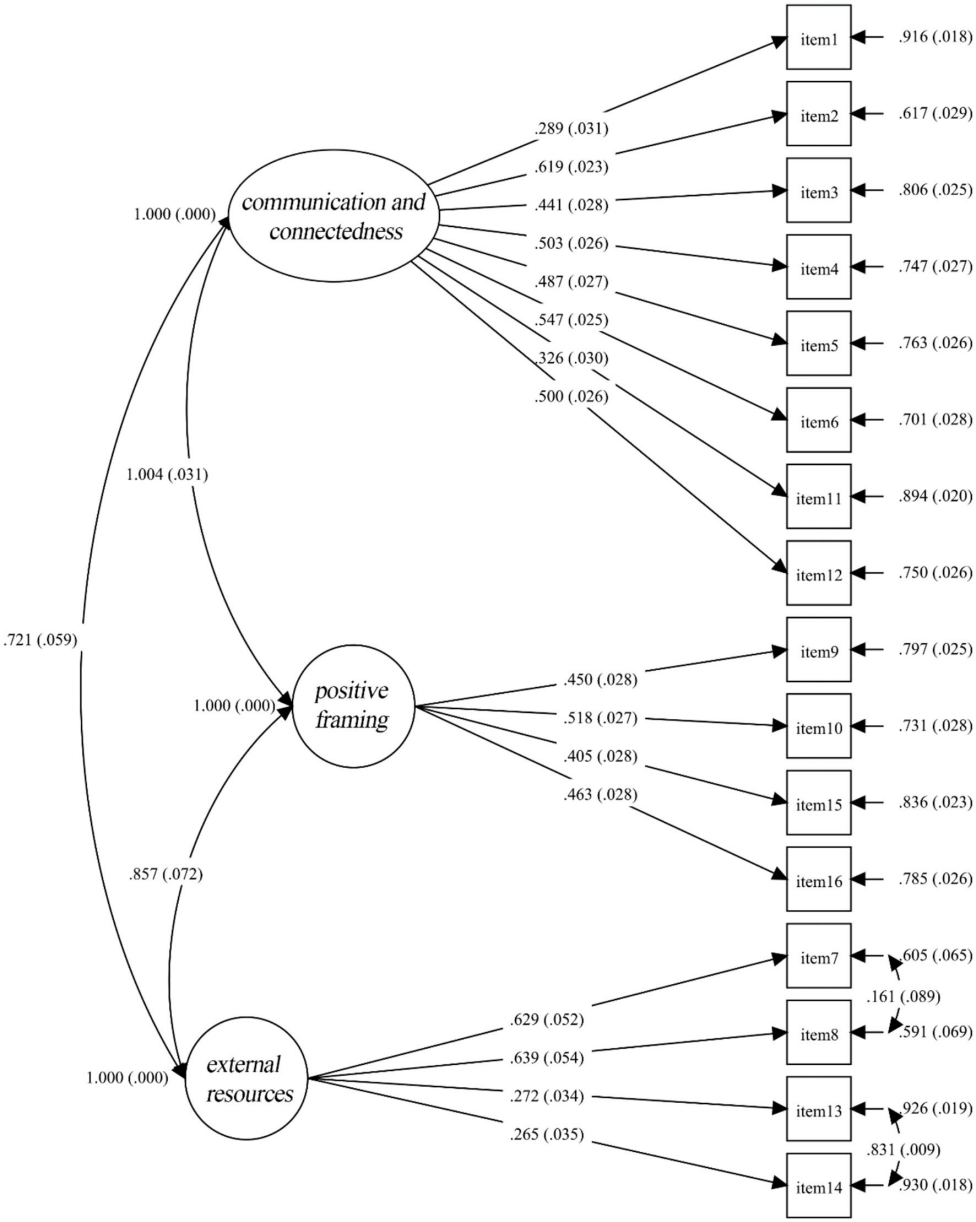
Factor structure and standardized factor loadings of FRS16_C.

Similar to Study 1, CFA was estimated with the maximum likelihood with the aid of Mplus 8.3. CFA results showed that the three-factor model demonstrated excellent model fit: χ^2^ (99) = 287.84, *p* <0.001; SRMR = 0.033; RMSEA = 0.041, CI = [0.036, 0.047]; TLI = 0.94; CFI = 0.95 (See Figure 2 for standardized factor loadings). The same set of alternative models were examined, as shown in Table 7, the fit indices of the one- and two-factor models were poor, whereas the five- and six-factor models showed parallel model fits to that of the proposed three-factor model. Considering the three-factor model has both more interpretable and parsimonious factor structure and five- and six-factor models did not show significant improvements in model fit, a holistic evaluation suggests a three-factor model should be adopted.

**Table 7:** Model fit indices of models with different number of factors.

**Model**	***df***	**χ^2^**	**CFI**	**TLI**	**RMSEA**	**SRMR**	**AIC**	**BIC**
One-factor	104	1766.62	0.589	0.526	0.119	0.074	31240.94	31482.60
Two-factor	103	877.01	0.809	0.777	0.081	0.096	30353.34	30600.02
Three-factor	99	287.84	0.953	0.943	0.041	0.033	29772.16	30038.99
Fix-factor	94	274.88	0.955	0.943	0.041	0.032	29769.20	30061.20
Six-factor	89	249.71	0.960	0.946	0.040	0.030	29754.04	30071.21

### Validity and Reliability of FRS16_C

#### Content Validity

As shown in Table 8, all items were significantly correlated to both its subscale and the full scale with a small to moderate strength (*r*s ≥ 0.31), demonstrating acceptable content validity.

**Table 8:** Item-to-scale and item-to-subscale correlations.

**Item**	**CC**	**PF**	**ER**	**FRS16_C**
1	0.446^**^			0.312^**^
2	0.649^**^			0.590^**^
3	0.543^**^			0.442^**^
4	0.543^**^			0.514^**^
5	0.558^**^			0.500^**^
6	0.597^**^			0.541^**^
11	0.540^**^			0.422^**^
12	0.580^**^			0.522^**^
9		0.653^**^		0.483^**^
10		0.691^**^		0.520^**^
15		0.572^**^		0.454^**^
16		0.628^**^		0.501^**^
7			0.547^**^	0.549^**^
8			0.590^**^	0.575^**^
13			0.839^**^	0.491^**^
14			0.835^**^	0.492^**^

** *p <0.01, CC, Communication and Connectedness; PF, Positive Framing; ER, External Resources; FRS16_C, Family Resilience Short Scale (Chinese)*.

#### Associations With Important Outcomes

*As* shown in Table 6, the full and subscales of FRS16_C were all significantly associated with proposed outcome variables in the expected directions.

#### Part-Whole Association

To our best knowledge, there are two Chinese versions of FRAS with three- (23) and five-factor models (24) which were validated with family caregivers and university students samples, respectively. Therefore, we would like to compare the performance of FRS16_C in comparison with other existing FRASs by examining the correlation coefficients of FRS16_C with the original FRAS, three-factor FRAS, and five-factor FRAS and compare their correlational patterns with demographic and related variables as a composite. As shown in Table 9, FRS16_C performed similarly to different versions of FRAS. Importantly, we observed that the shortened FRS16_C retained at least 70% of the predictive value of the 54-item scale. This pattern indicated that, given its brevity, the 16-item scale developed in the present study can stand in for the full 54-item scale in terms of predictive value.

**Table 9:** Correlations of FRASs and FRS16_C with demographic and outcome variables.

	**FRS16_C**		**FRAS**		**3-factor FRAS**		**5-factor FRAS**	
			**(** **16** **)**		**(** **23** **)**		**(** **24** **)**	
	***r***	***p***	***r***	***p***	***r***	***p***	***r***	***p***
Age	0.180	<0.001	0.206	0.002	0.175	0.010	0.186	0.006
Annual household income	0.184	<0.001	0.272	<0.001	0.230	0.001	0.242	<0.001
Education level	0.042	0.155	0.120	0.081	0.119	0.084	0.103	0.135
Quality of life	0.589	<0.001	0.694	<0.001	0.687	<0.001	0.689	<0.001
General health	−0.549	<0.001	−0.624	<0.001	−0.571	<0.001	−0.620	<0.001
Family cohesion	0.410	<0.001	0.439	<0.001	0.455	<0.001	0.399	<0.001
Relationship satisfaction	0.437	<0.001	0.554	<0.001	0.525	<0.001	0.512	<0.001
Perceived community support	0.566	<0.001	0.578	<0.001	0.546	<0.001	0.598	<0.001

#### Reliability

The Cronbach's alphas of Communication and Connectedness, Positive Framing, and External Resources were 0.67, 0.51, and 0.68, respectively. The reliability of overall FRS16_C was 0.78 and the test-retest reliability of FRS16_C in 2-weeks interval was acceptable (ICC = 0.87).

### Measurement Invariance of FRS16

Since the group-level CFA test results were found satisfactory with both the US and Chinese samples separately, configural invariance was established. Thus, the three-factor structure is valid for both groups (47). A multi-group three-factor model was still tested with no parameter constraints, as shown in Table 10, the fit indices of this baseline model were satisfactory, which showed converging support for configural variance. Next, we tried to establish metric variance by running a more stringent multi-group model with the factor loadings coefficients constrained to be equal across groups on top of the baseline model. The multiple-group model showed satisfactory fit (see Table 10), both ΔCFI and ΔRMSEA were below the recommended cut-offs [ΔCFI ≤ 0.010 and ΔRMSEA ≤ 0.015; (48)], suggesting that respondents attributed the same meaning to the latent factor (47).

**Table 10:** Evaluations of measurement invariance.

	**χ2**	***df***	***p***	**CFI**	**TLI**	**RMSEA**	**ΔCFI**	**ΔRMSEA**
Configural invariance	986.989	198	<0.001	0.914	0.896	0.058		
Metric invariance	1018.309	211	<0.001	0.912	0.900	0.057	−0.002	−0.001

## General Discussion

To provide a measure of family resilience for contexts in which assessment time is relatively limited, we abbreviated the Family Resilience Assessment Scale [FRAS (16)] to a 16-item version, the FRS16. The proposed 3-factor model was supported across the US and Chinese samples. The 3-factor model consists of easily interpretable factors that cover the most essential components of family resilience, namely, ***Family Communication and*** ***Connectedness***, ***Positive Framing***, and ***External Support*** (4, 28, 49, 50). These three factors also cover all of the subprocesses in Walsh (28)'s theory and correspond to the different family adaptive subsystems (maintenance system, meaning system, and ecosystem) discussed in Henry et al. (34). Family communication and connectedness signifies the way through which the family members organize and communicate in response to external stressors. It captures the core elements of the maintenance system. Meanwhile, the positive framing factor involves the family world view and the ability to maintain positive strength-based outlook. This factor represents the adaptability of the family meaning system. Finally, the external resources capture positive features and resources in the external ecosystem in which crises and opportunities occur.

We also noticed that the factor loadings of the items related to family spirituality (e.g., item 13: we attend church/synagogue/mosque services) were lower (<0.30) than the two other items about social support (>0.60) in the Chinese sample. Comparatively, the factor loadings of these four items were more homogenous in the US sample. Li et al. (24) suggested that religious support is less significant in Chinese societies. In Chinese culture, the practice of ancestor worship is considered as the most common form of spiritual activity. In a study conducted in Singapore, Chinese participants identified ancestors as their spiritual sources (51), yet prominence and the format of these spiritual activities are diverse even within the Chinese culture. This might explain the more heterogeneous factor loadings of this subscale in the Chinese sample. Since the factor loadings were still statistically significant, and the item-to-subscale and item-to-total correlations remain high, we decided to keep these two items in the External Resources subscale. Nevertheless, future research should further explore the value of religious and spiritual activities in Chinese family resilience.

To assess the psychometric equivalence of the FRS16 across the US and Chinese samples, measurement invariance analyses were conducted. The configural invariance suggested that the basic organization (the 3-factor structure) is supported in both cultures while the metric invariance suggested that the contribution of each item to the latent factor to a similar degree across the two samples. These findings suggested that the proposed FRS16 includes the most essential items that measure the common, fundamental processes of family resilience across cultures. Thus, it is a viable tool for assessing family resilience in cross-cultural studies especially when assessment time is limited. Nevertheless, if cultural sensitivity is the major concern of the research, researchers can consider other longer versions which were developed specifically for a single culture [Chinese: (24)]. We also examined the performance of the FRS16 in comparison with three other versions of FRAS including the full 54-item version. The part-whole correlation between the FRS16 with the original FRAS reaches 0.87. We also observed strong correlations between the FRS16 with other adapted versions of the scale (*rs* >0.77). Additionally, the FRS16 shares comparable associations with validation instruments with FRAS in three different lengths.

In both samples, the FRS16 demonstrated good internal consistency for the total scale (αs > 0.80). The reliabilities of all subscales were satisfactory except Positive Framing in the Chinese sample. Nevertheless, despite the relatively low Cronbach's α (0.51), the item-to-subscale, and item-to-total correlations were both high and significant. The four items in the Positive Framing subscale were also strongly loaded on the expected latent factor. Given that this subscale consists of only four items that were related to two different types of positive framing—showing high efficacy in problem solving (e.g., Item 10: We can survive if another problem comes up) and normalizing stressors and crises (e.g., Item 15: We accept stressful events as a part of life), the relatively lower alpha was considered acceptable.

The present studies indicated that the FRS16 and its three subscales were all significantly associated with proposed outcome variables (i.e., quality of life, general health, family cohesion, relationship satisfaction, perceived community support) in the expected directions for both the US and Chinese samples. It is noted that the External Resources subscale showed relatively lower correlations with relationship satisfaction and family cohesion in both samples. It might suggest that the ability to utilize external and internal resources to cope with crisis were comparatively less crucial to the cohesion and relationship satisfaction of a family compared to Communication and Connectedness and Positive Framing. However, it does not in any way imply that External Resources was a less important component of family resilience, as it demonstrated correlation with other validation instruments in similar sizes as other components, such as its associations with general health and perceived community support.

The present study has a few limitations. First, the study was conducted on online crowdsourcing samples. Although crowdsourcing data enjoys benefits such as the more diversified demographic background of participants (52), it still cannot represent the general population. This concern has also been reflected in our US sample with higher proportion of male, and in Chinese sample with majority coming from high-annual-household-income family. Efforts should be made in validating the FRS16 with more representative and diverse samples. Additionally, some researchers argued that crowdsourcing data has the problem of cross-contamination and selective dropouts, but these issues are more relevant to experimental studies (53). Afterall, precaution is still needed in interpreting the results obtained from these crowdsourcing platforms. Second, the present study did not focus on populations that are facing acute or chronic stressors and traumatic events (e.g., caregiver of a family member who has chronic illness, recent death of a family member, economic hardship) or populations with different family characteristics (e.g., the number of family members cohabiting). The importance and meaning of family resilience could be different for people who are encountering crises or with different family characteristics. Future research could test the reliability and validity of the FRS16 in diverse populations. Third, we used quality of life, general health, relationship satisfaction, family cohesion, and perceived community support for validating the FRS16. These variables might not fully reflect the three proposed processes in family resilience. Future research might include more theoretically relevant outcome measures such as family and community support [Social Support Index; (54)]. Forth, given that the cross-sectional nature of current studies, the directions of associations between FRS16 and validation instruments remain unknown, future studies are needed to provide support for predictive validations with a longitudinal or experimental design. Fifth, as the measurements were self-reported, responses might be contaminated by social desirability. However, respondents' anonymity is underscored in the beginning of the survey to minimize the influence of social desirability. Lastly, only individual family member's perception of the family dynamics and resilience was captured in this study. Other family members' perceptions may vary. In other words, it might not reflect family resilience comprehensively, as it fails to capture this process positioning families as a unit. Nevertheless, validating a short version of family resilience scale is a starting point to “study resilience in the family as a functional unity” (55) by making the measurement of each family members' perceptions less time-consuming.

Overall, the present study contributes to the literature by developing a short form of family resilience scale and cross-validated it in Western and Eastern cultures. The short version captures the essential items and the most fundamental processes underlying family resilience. Given its brevity, the FRS16 possesses acceptable psychometric properties in both cultures. It is suitable for cross-cultural study and research in which the testing time is limited such as therapy outcome assessment, intensive longitudinal research, and telephone survey.

## Data Availability Statement

The raw data supporting the conclusions of this article will be made available by the authors, without undue reservation.

## Ethics Statement

The studies involving human participants were reviewed and approved by Human Research Ethics Committee of the Hong Kong Shue Yan University. The patients/participants provided their written informed consent to participate in this study.

## Author Contributions

TC contributed to study conceptualization, survey design, leading statistical analyses and interpretation of results, and writing the original text. CT contributed to study conceptualization, survey design, results interpretation, administrating and supervising the project, providing comments to revise the manuscript, and refine the data analysis. TS facilitated the data analysis and contributed to reviewing and editing the manuscript. HK provided study measures and contributed to the survey development and refinement. All authors contributed to the article and approved the submitted version.

## Funding

This study was funded by a matching research grant awarded to the Wan Chow Yuk Fan Centre for Interdisciplinary Evidence-based Practice & Research at the Hong Kong Shue Yan University UL/RS/2021/001.

## Conflict of Interest

The authors declare that the research was conducted in the absence of any commercial or financial relationships that could be construed as a potential conflict of interest.

## Publisher's Note

All claims expressed in this article are solely those of the authors and do not necessarily represent those of their affiliated organizations, or those of the publisher, the editors and the reviewers. Any product that may be evaluated in this article, or claim that may be made by its manufacturer, is not guaranteed or endorsed by the publisher.

## Footnotes

^1^See https://help.credamo.com/web/#/4?page_id=118 for a list of publications in 2020 using Credamo to collect data.

^2^The categorization of annual household income in 2019 set by National Bureau of Statistics of China was as follow: the lowest 20% cutoff was 7380.4 CNY, following by 15777.0 CNY (mid low), 25034.7 CNY (mid), 39230.5 CNY (upper mid) and 76400.7 CNY (high). See http://www.stats.gov.cn/tjsj/ndsj/2021/indexeh.ht.
