# Supplementary material for: Family Resilience Scale Short Form (FRS16): Validation in the US and Chinese Samples
Source: Front Psychiatry. 2022 May 13;13:845803. doi: 10.3389/fpsyt.2022.845803 (PMC9136042; doi:10.3389/fpsyt.2022.845803)
Supplement: Supplementary file 1 [file Table_1.pdf]

Appendix A –FRS16 and proposed three-factor structure

	从您的角度，如何描述你的家庭/家庭成员？<br>How would you describe your family/family members?	*Strongly disagree*	*Disagree*	*Agree*	*Strongly agree*
	**Communication and Connectedness**				
1	We can compromise when problems come up.<br>当问题出现时，我们可以妥协。	1	2	3	4
2	We can talk about the way we communicate in our family.<br>我们可以讨论家庭中沟通的方式。	1	2	3	4
3	We consult with each other about decisions.<br>我们会在作出决定时咨询大家。	1	2	3	4
4	We define problems positively to solve them.<br>为了解决事情，我们正面地理解问题。	1	2	3	4
5	We discuss problems and feel good about the solutions.<br>我们会就问题作出讨论，亦对解决方案有良好的感觉。	1	2	3	4
6	We discuss things until we reach a resolution.<br>我们会就问题作出讨论，直到达到解决方案。	1	2	3	4
11	We will not be taken for granted by family members.<br>我们的家庭成员不会认为我们的付出是理所当然的。	1	2	3	4
12	We often listen to family members concerns or problems.<br>我们时常聆听家人的担忧或问题。	1	2	3	4
	**Positive Framing**				
9	We can solve major problems.<br>我们可以解决重大问题。	1	2	3	4
10	We can survive if another problem comes up.<br>当有其他问题出现时，我们仍可以应付。	1	2	3	4
15	We accept stressful events as a part of life.<br>我们接受压力的事件的发生，是生活的一部份。	1	2	3	4
16	We accept that problems occur unexpectedly.<br>我们接受突如其来的问题。	1	2	3	4
	**External Resources**				
7	We feel people in this community are willing to help in an emergency.<br>我们觉得社区内的人会愿意在危急时伸出援手。	1	2	3	4
8	We know there is community help if there is trouble.<br>我们知道当我们有麻烦时，社区会伸出援手。	1	2	3	4
13	We attend church/synagogue/mosque services.<br>我们会出席社区/宗教活动。	1	2	3	4

14	We participate in church activities.<br>我们参与**籌備及執行社**区/教会活动。	1	2	3	4
